# A Life-Changing Diagnosis of Focal Segmental Glomerulosclerosis in a Young Hispanic Male

**DOI:** 10.7759/cureus.20323

**Published:** 2021-12-10

**Authors:** Aboud Kaliounji, Kristen Farraj, Rasna Juta, Zae Kim

**Affiliations:** 1 Internal Medicine, St. George's University School of Medicine, New York City, USA; 2 Internal Medicine, Nassau University Medical Center, East Meadow, USA; 3 Nephrology, Nassau University Medical Center, East Meadow, USA

**Keywords:** kidney biopsy, human immunedeficiecy virus (hiv) infection, end stage renal disease (esrd), nephrotic, focal segmental glomerular sclerosis

## Abstract

Focal segmental glomerulosclerosis is a nephrotic syndrome characterized by significant proteinuria on urinalysis and sclerosis in parts of at least one glomerulus on biopsy. While primary cases are on the rise over the past two decades, it is important to rule out etiologies that cause secondary focal segmental glomerulosclerosis such as HIV and Hepatitis B. The prevalence of this disease over the past few decades has been notably higher in men and in particular African Americans. Here, we discuss a case of a 25-year-old Hispanic man who was found to have focal segmental glomerulosclerosis after initially presenting with facial and upper and lower extremity swelling.

## Introduction

In the United States, focal segmental glomerulosclerosis (FSGS) is the most common glomerular cause of end-stage renal disease (ESRD) [1]. FSGS refers to a histologic finding that accounts for almost 40% of the nephrotic syndrome (NS) cases in adults and 20% in children [1]. It is characterized by the presence of sclerosis in parts (segmental) of at least one glomerulus (focal) in the entire kidney biopsy, when examined by light microscopy (LM), immunofluorescence (IF), or electron microscopy (EM). While marked proteinuria and podocyte injury are also characteristics of this disease, it is critical to eliminate other systemic or renal diseases that may result in similar presentations.

The prevalence of FSGS associated with ESRD has gradually increased over the past decades. While absolute incidence and prevalence are difficult to obtain, recently published literature reported that the annual incidence rates ranged from 0.2 to 1.8 per 100,000 population per year [1]. Among these cases, there is a significant ethnic and racial predisposition. The incidence rate of FSGS tends to be 1.5 higher in men than in women. More specifically, black patients have a fourfold higher risk of developing ESRD compared to white and Asian patients.

FSGS can be classified as either primary or secondary. Primary FSGS is believed to be secondary to toxins circulating permeability factors that cause podocytopathy. On the other hand, secondary FSGS refers to an adaptive phenomenon in which the number of functional nephrons is reduced. This can be explained by genetic components, drugs, or virus-associated illnesses [2]. Thus, patients are considered to have primary FSGS once secondary causes are ruled out such as Hepatitis B, HIV, sickle cell disease, Parvovirus, hypertension nephropathy, renal dysgenesis, oligomeganephronia, cholesterol emboli, lymphoma, Alport’s syndrome, radiation nephritis, drug-related causes, and/or familial podocytopathies. Here, we describe a case of primary FSGS in a young Hispanic male.

## Case presentation

A 25-year-old Hispanic male with no known past medical history presented to the Emergency Department (ED) with a two-week history of swelling in his face, hands and feet as well as hematemesis containing blood clots for five days. He also had nausea and diarrhea. The patient reported a non-productive cough for one year for which he had been intermittently self-medicating with amoxicillin 500 mg tablets from El Salvador and Tylenol with no improvement in symptoms. He also presented to the ED two weeks prior complaining of cough, subjective fevers and pleuritic chest pain and received Toradol 60 mg intramuscularly for diagnosis of “chest wall pain.” At that time, his vital signs were as follows: blood pressure (BP) of 148/84 mmHg; heart rate (HR) of 89 bpm; SpO_2_ of 98% and a temperature of 98F. He was discharged from the ED with a prescription for Naprosyn 500 mg oral one tablet every 12 hours as needed for 14 days. Social history revealed many prior tattoos and social alcohol use (4-5 beers on the weekend), no prior tobacco, illicit or IV drug use. He reported safe sexual practices using condoms for sexual intercourse with females. Family history was significant for type 2 diabetes mellitus in his mother and a maternal aunt with stage 3 chronic kidney disease secondary to diabetic nephropathy.

The patient returned to the ED two weeks later complaining of hematemesis, new onset edema, increasing lethargy, increased appetite, nausea and frequent loose bowel movements. He denied any abdominal or flank pain and subjective fevers. The patient reported a noticeable decrease in urination but could not recall the onset. Vital signs were as follows: BP of 165/85, HR of 113 bpm, SpO_2_ of 97% on room air and a temperature of 98F. Physical examination was significant for 2-mm pitting edema of the hands and lower extremities to the mid-shin and dried bright red blood in his oral cavity with no evidence of ongoing bleeding. Laboratory studies were significant for profound anemia with a hemoglobin of 5.7 g/dL and acute renal failure with a creatinine of 24.2 mg/dL, calculated GFR of 3 and a urinalysis showing +4 proteinuria with a urine protein-creatinine ratio 10,000 mg/g. Desmopressin was administered for the recent bleeding in the setting of uremia and the patient was transfused with packed red blood cell units to increase his hemoglobin above 7 g/dL. He also received labetalol 20 mg IVP for hypertensive urgency. Gastroenterology and nephrology were consulted and the patient was admitted to inpatient medicine service for continued evaluation and treatment.

**Figure 1:**
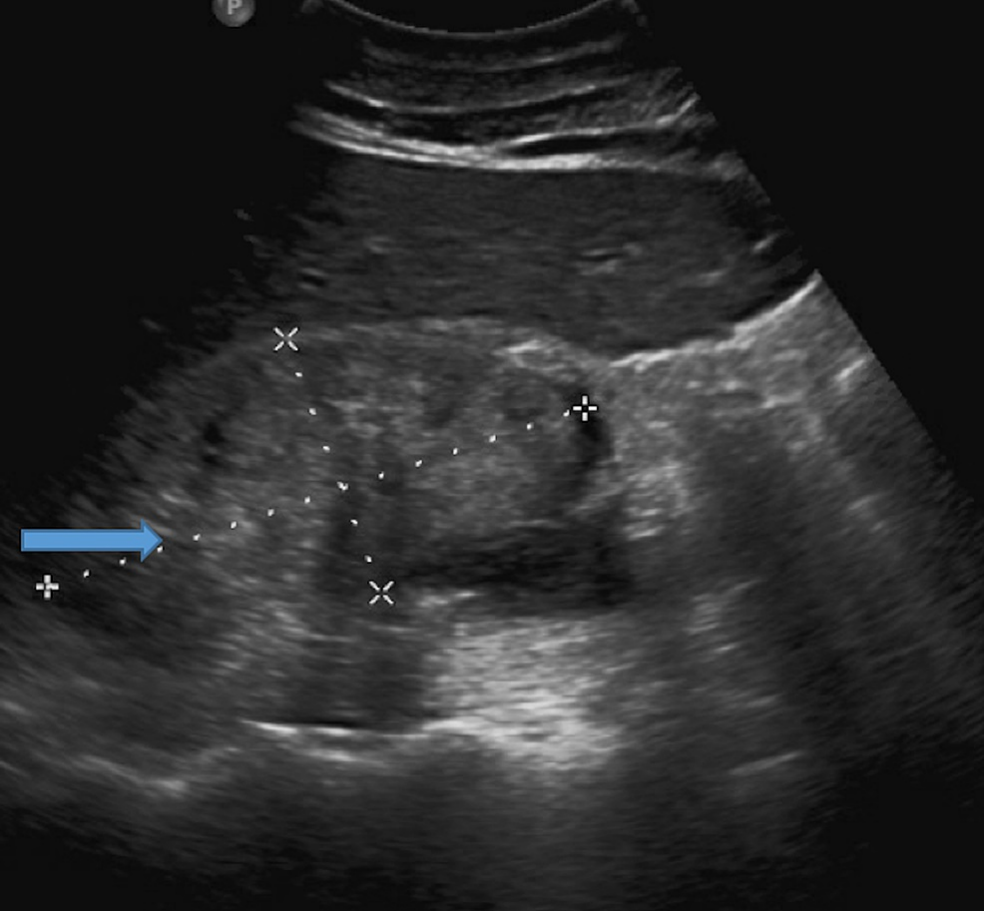
Ultrasound of the right kidney (blue arrow) measuring 9.61 by 4.61 cm.

**Figure 2:**
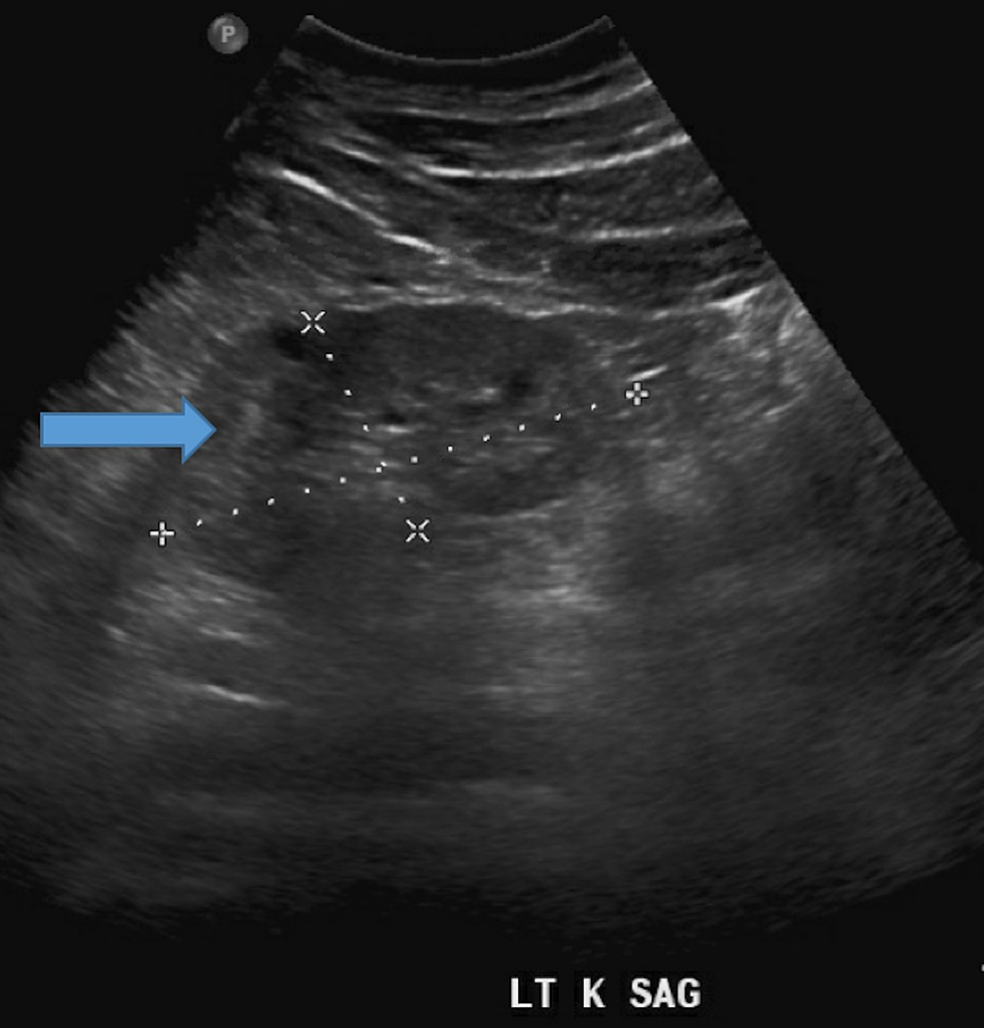
Ultrasound of the left kidney (blue arrow) measuring 8.67 by 4.09 cm. Left kidney (LT K), Saggital (SAG)

**Figure 3:**
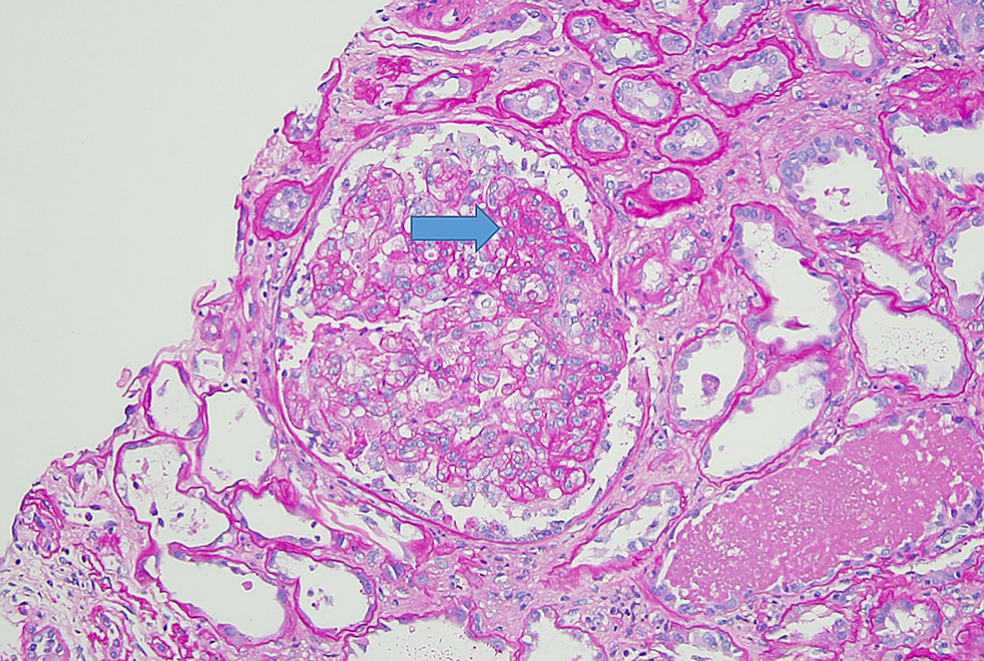
H&E stain of a renal biopsy showing segmental sclerosis (arrow) indicative of focal segmental glomerulosclerosis. Hematoxylin and Eosin (H&E)

**Figure 4:**
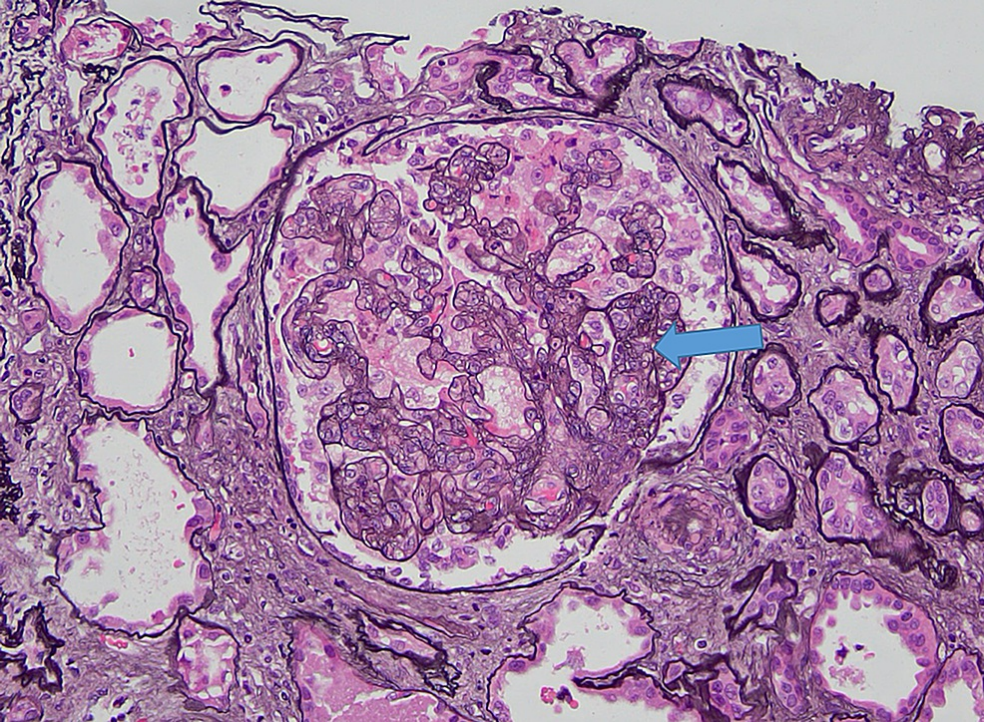
JMS stain of a renal biopsy revealing severe collapsing glomerulopathy with advanced tubulointerstitial scarring. Jones' Methenamine Silver (JMS)

**Figure 5:**
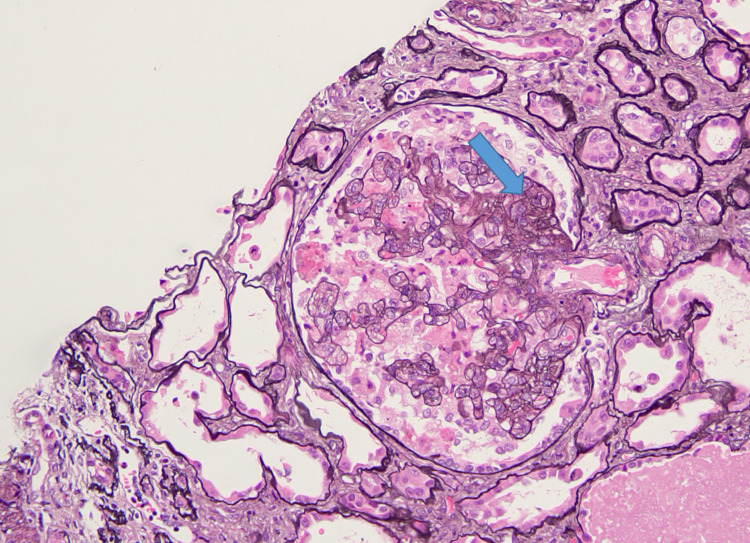
Renal biopsy stained with JMS showing focal segmental sclerosis. Jone's Methenamine Silver (JMS)

Kidney ultrasound revealed severely increased echogenicity and enlargement bilaterally. The right and left kidneys measured 9.61 by 4.61 cm and 8.67 by 4.09 cm, respectively (Figures 1, 2). Kidney biopsy findings revealed severe collapsing glomerulopathy, a subtype of FSGS with advanced tubulointerstitial scarring and mild to moderate diffuse arterio- and arteriolosclerosis (Figures 3-5). There was no increase in immune complex deposition on renal biopsy. Laboratory evaluation for infectious, autoimmune and vasculitis including HIV, Hepatitis B and C, ANA, double-stranded DNA, MPO PR3 and anti-GBM were negative.

During hospitalization, the patient’s BP was difficult to control with a systolic blood pressure persistently in the 140-150 mmHg range while receiving three antihypertensives: labetalol 100 mg one tablet orally twice daily, Norvasc 5 mg one tablet orally once daily and hydralazine 25 mg one tablet orally three times daily.

**Figure 6:**
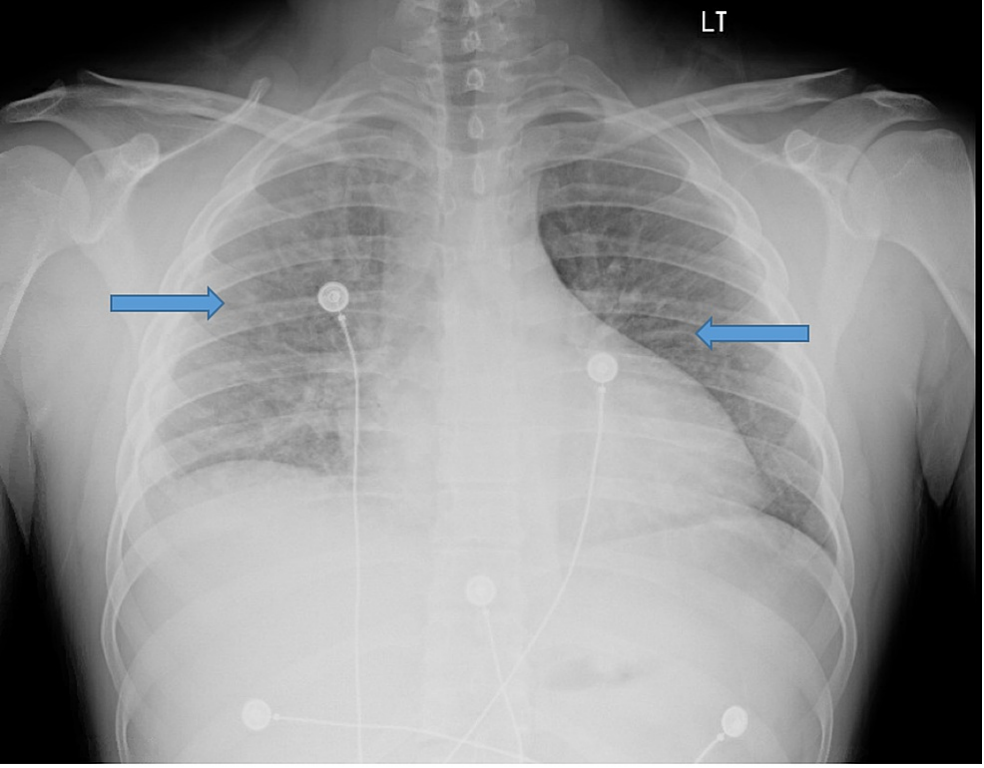
Chest x-ray, AP view, showing increased interstitial lung markings bilaterally. Anteroposterior (AP)

Chest x-ray showed increased bilateral interstitial lung markings and computerized tomography (CT) of the thorax was recommended for further evaluation (Figure 6). He was treated for suspected pneumonia in the setting of a chronic cough and new-onset fevers during admission.

The metabolic acidosis and hyperkalemia were refractory to medical therapy and it was determined that the patient's kidney damage was irreversible, requiring hemodialysis. He was started on sevelamer for hyperphosphatemia. Interventional radiology (IR) was consulted to place a tunneled hemodialysis catheter in the right internal jugular vein. The patient underwent hemodialysis every two days with improvement in BUN/Cr to 42/9.8 two weeks later.

**Figure 7:**
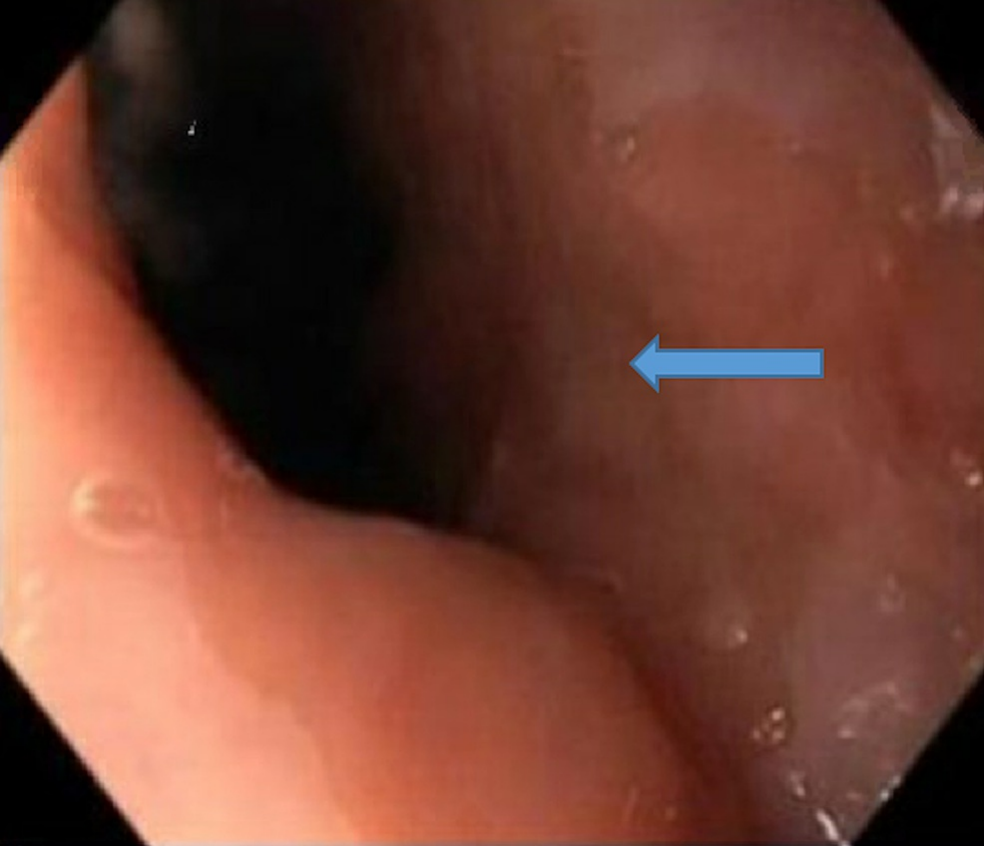
EGD showing small hiatal hernia. Esophagogastroduodenoscopy (EGD)

**Figure 8:**
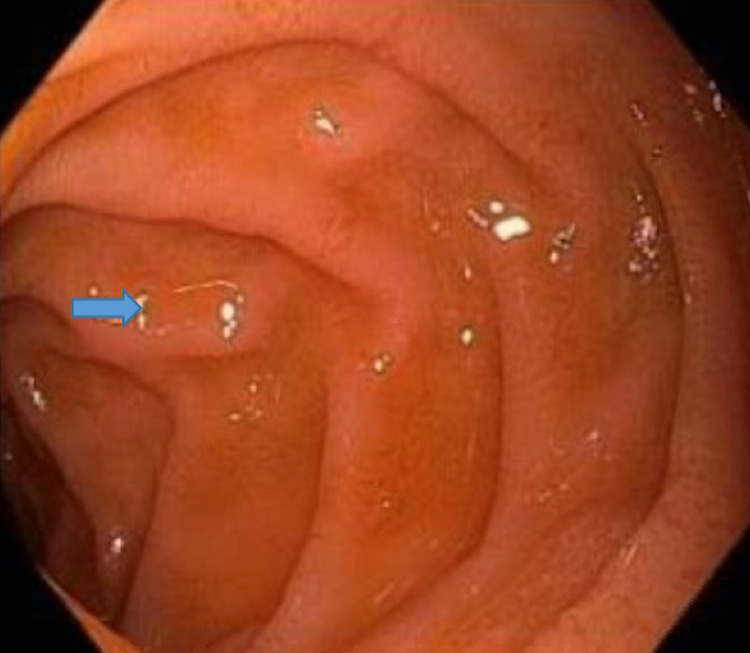
EGD showing prominent Brunner's glands in the second portion of the duodenum. Esophagogastroduodenoscopy (EGD)

**Figure 9:**
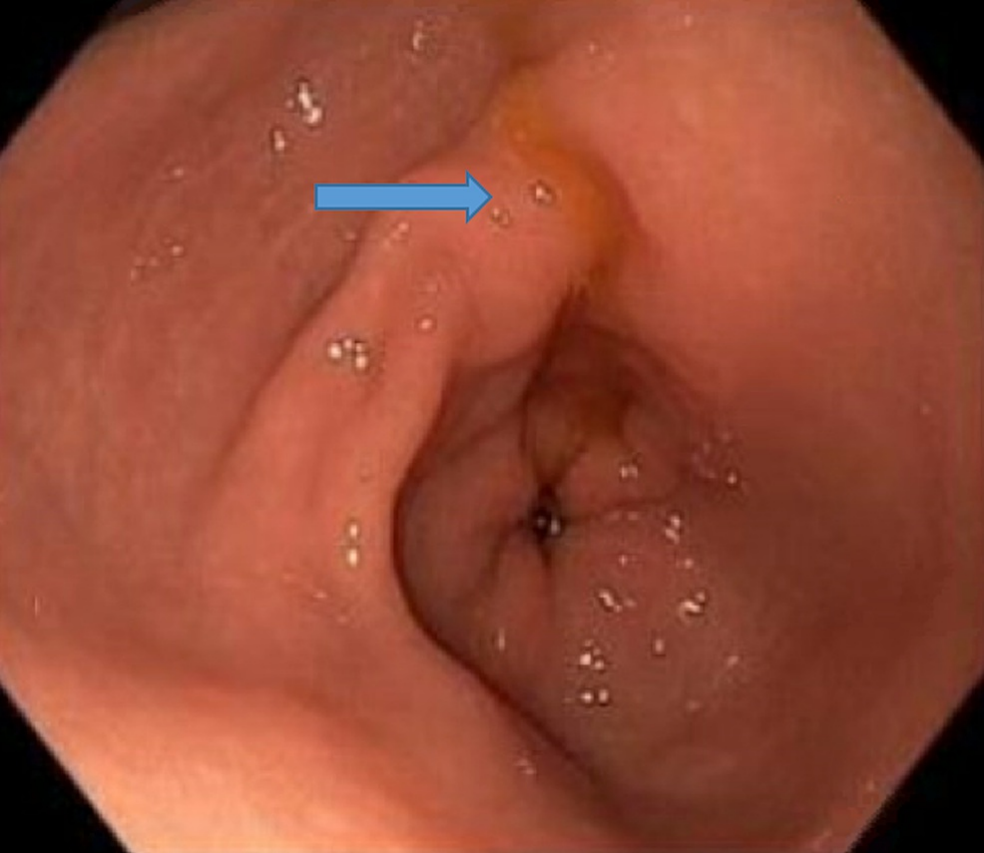
EGD showing erythematous mucosa in the antrum of the stomach. Esophagogastroduodenoscopy (EGD)

The patient responded to the transfusions appropriately with no further evidence of gross bleeding and later was started on darbepoetin alfa (Aranesp) with hemodialysis. Gastroenterology performed an esophagogastroduodenoscopy (EGD) that showed a small hiatus hernia, prominent Brunner's glands in the duodenum and erythematous mucosa in the antrum and body of the stomach (Figures 7-9). The colonoscopy was unremarkable except for non-bleeding internal hemorrhoids.

After two weeks of admission, the patient was discharged home with scheduled hemodialysis and a specialist follow-up.

## Discussion

FSGS is characterized by a pattern of segmental glomerular scars with the clinical findings of proteinuria. The prevalence of primary FSGS is on the rise and now makes up one-third of the NS cases among adults and half of the NS cases in African American (AA) individuals, in whom it is seen more commonly. However, here we present a case of rapidly progressive FSGS in a young Hispanic male.

FSGS is commonly seen among AAs with a higher rate seen in men with a mean age ranging between 23 and 57 years old [3]. An increased risk of acquiring FSGS among AAs has been seen with polymorphic variants (G1 and G2) in the Apolipoprotein 1 (APOL-1) gene [4,5]. Proteinuria, high creatinine, elevated blood pressure, and NS are some of the hallmarks of this disease [5]. Although our patient had both clinical and laboratory findings consistent with NS, an IR-guided renal biopsy was performed to confirm FSGS. Pathology showed focal and segmental destruction of capillary tufts with an increased matrix. Classically, FSGS is treated with high-dose prednisone along with calcineurin inhibitors in patients with resistant FSGS.

In the United States, the annual incidence of ESRD cases secondary to FSGS is 7 per million for the general population, 20 per million for AAs, and 5 per million for white individuals. Cases of ESRD due to acquired immune deficiency syndrome (AIDS) nephropathy were increasing at a fast rate until 1995 and plateaued thereafter [6]. The causes for the recent spike in the incidence of idiopathic FSGS cases are complex, but likely reflect a true rise in the prevalence of FSGS over the past two decades. Epidemiologic data from the United States Renal Data Systems (USRDS) show that the incidence of ESRD secondary to primary FSGS has increased substantially, both as absolute numbers and as a fraction of the total ESRD incident population. FSGS now accounts for 3.3% of ESRD cases. A study conducted by Dragovic et al. found that the increase has occurred among both white, black and Hispanic patients across the board and that FSGS was most prevalent in patients greater than or equal to 45 years of age [2]. This is important since our patient was only 26 years old at the time of diagnosis. Another single-center study conducted on 65 patients found that FSGS was more prevalent in AA than in non-black patients [7]. AAs accounted for roughly 45% of all cases. They, again, found that FSGS was more common in patients of older age.

There was one previous case series conducted in Colombia in which a total of 1,185 native renal biopsies were examined over a 9.5-year period. The results of this study found that FSGS was still the most prevalent glomerulopathy among their Hispanic population and represented 34.8% of primary glomerulopathies [8]. This is significant as it is close to what has been reported in other studies in reference to AA patients. This further supports the importance of our case and the need for increased awareness among clinicians of the rise in prevalence of FSGS among other ethnic groups besides AAs. 

Other variants have been reported in addition to the classic focal and segmental scarring: endocapillary hypercellularity, collapsing glomerulopathy with segmental or global glomerular collapse associated with a rapid decline in renal function [9]. Poor prognosis is associated with renal insufficiency, AA race and nephrotic range proteinuria, with half of the patients experiencing renal failure in six-eight years. While many studies have shown that GI bleeding can be a complication of severe kidney failure, our patient’s presentation which includes hematemesis raises the importance of further investigating the prevalence of upper GI bleed in FSGS patients.

## Conclusions

In summary, FSGS is a podocytopathy due to a scarring process such as circulating factors, drug use and infection. Primary FSGS may quickly progress to end-stage kidney disease. While the majority of cases are found in AA men, it is crucial to further investigate patients who present with risk factors to prevent the progression of the disease. This study provides a contribution toward understanding the increasing rise of focal segmental glomerulonephritis among the Hispanic population, with potential implications for future research.
